# Using Yeast Two-Hybrid Screening and Structural Modeling to Identify Candidate Hrr25 Kinase Interactors at the Meiotic Kinetochore in *Saccharomyces cerevisiae*

**DOI:** 10.3390/ijms27094083

**Published:** 2026-05-02

**Authors:** Meenakshi Agarwal, Sankalpa Chakraborty, Santanu K. Ghosh

**Affiliations:** 1 Department of Bioscience and Bioengineering, Indian Institute of Technology, Bombay, Powai, Mumbai 40076, India; 2 Department of Biological Sciences, Florida State University, Tallahassee, FL 32306, USA

**Keywords:** *Saccharomyces cerevisiae*, Hrr25, yeast two-hybrid, kinetochore, mono-orientation, meiosis, phosphorylation, restriction mapping, structural modeling

## Abstract

In *Saccharomyces cerevisiae* (*S. cerevisiae*), sister kinetochores are mono-oriented during meiosis I to ensure accurate homolog segregation, a process dependent on Hrr25 kinase activity. However, its direct interactors remain poorly defined. To address this, we performed a yeast two-hybrid (Y2H) screen using Hrr25 as bait. *HRR25* was cloned into a Y2H vector and functionally validated by complementation of a temperature-sensitive *hrr25-ts* mutant. Screening across three reading frames identified three putative interactors: Hed1, Cyr1, and Rep1. Additional open reading frames (ORFs), including *DAD1*, *SYS1*, and *YDR015C* were identified but were oppositely oriented to the GAL4 activation domain. Structural modeling and phosphorylation prediction identified high-confidence Hrr25 target residues, including S70/T73 on Hed1, S323 on Rep1, and S198/S527 on Cyr1, whereas Sys1 and YDR015C lacked favorable sites. Although Dad1 was not validated as a direct interactor from Y2H, S63 was identified as a favorable phosphorylation site, and its full-length ORF in the interacting clone and known biological role supported its inclusion. Among the meiotic candidates, Hed1 may link Hrr25 activity to homologous recombination, while Dad1 represents a plausible target for regulating kinetochore–microtubule interactions. Collectively, these findings identify new candidate interactors and substrates of Hrr25 and suggest a broader role in coordinating recombination and kinetochore function during meiosis, warranting further experimental validation.

## 1. Introduction

Accurate chromosome segregation during meiosis is essential for maintaining euploidy across generations. Errors in this process can have serious consequences, including aneuploidy, which may result in infertility, cancer, and congenital disorders such as Down syndrome [1,2,3]. Similarly to mitosis, meiotic chromosome segregation is controlled by the kinetochore, a large multiprotein complex that assembles at the centromere of a chromosome and provides attachment sites for microtubules, thereby linking chromosome movements to microtubule dynamics [4,5,6]. However, meiosis involves additional layers of complexity and relies on four fundamental and conserved events: (1) homologous chromosomes pair and undergo recombination to form chiasmata, which physically link non-sister chromatids; (2) sister kinetochores of each homolog orient toward the same spindle pole (mono-orientation), enabling proper bi-orientation of homolog pairs on the meiosis I (MI) spindle; (3) cohesin is released in a stepwise manner during meiosis I and II to facilitate orderly chromosome segregation; and (4) suppression of DNA replication prior to second meiotic division [7].

In *Saccharomyces cerevisiae* (*S. cerevisiae*), the mono-orientation of sister kinetochores during meiosis I is directed by the monopolin complex, comprising four proteins: Mam1, Csm1, Lrs4, and the kinase Hrr25. This complex is specifically recruited to the centromere during prophase I, where it promotes monopolar attachment of sister kinetochores to the spindle microtubules during meiosis I [8,9]. Hrr25, a 494-amino acid multifunctional casein kinase I family member, has a well-characterized N-terminal kinase domain and a C-terminal proline/glutamine (P/Q)-rich region [10]. It is essential for cell viability and exerts several functions during vegetative growth. Mutations in *HRR25* result in slow cell proliferation and increased sensitivity to DNA damage [11]. During meiosis, a kinase-dead mutant of Hrr25 results in abnormal bi-orientation of sister chromatids at metaphase I and produces defective spores with unequal DNA content, highlighting the necessity of its kinase activity during meiosis I [12].

Despite its established importance, the full repertoire of meiotic substrates of Hrr25 remains poorly defined. Structural and biochemical studies suggest that the monopolin core proteins Csm1 and Lrs4 form a V-shaped complex that functions as a molecular clamp. In association with Mam1, this complex interacts with the N-terminal region of Dsn1, a component of the MIND kinetochore complex, thereby facilitating the physical crosslinking of sister kinetochores at meiosis I [13]. Mam1 has also been shown to recruit Hrr25 to this monopolin–kinetochore interface, positioning the kinase in proximity to potential substrates such as Dsn1. It has been proposed that Hrr25-mediated phosphorylation of the N-terminal region of Dsn1 may regulate the affinity or stability of monopolin binding at kinetochores [14]. However, direct in vivo evidence supporting Dsn1 as a bona fide Hrr25 substrate is currently lacking, and specific phosphorylation sites within Dsn1 have not been conclusively identified [15]. Moreover, the regulatory contribution of Dsn1 phosphorylation appears to be more closely linked to modulation of kinetochore architecture rather than direct physical tethering.

Although Mam1 itself has been identified as a phosphorylation target of Hrr25, this modification is dispensable for establishing mono-orientation during meiosis I [12]. Additionally, Hrr25, in coordination with the Dbf4-dependent Cdc7 kinase, phosphorylates Rec8, a meiosis-specific cohesin subunit. This phosphorylation is required for Rec8 cleavage by separase during the transition from metaphase I to anaphase I [16]. Nonetheless, there is currently no evidence that Rec8 phosphorylation by these kinases directly contributes to the establishment of sister chromatid mono-orientation.

These findings suggest that additional kinetochore or chromatin-associated proteins may serve as critical Hrr25 substrates, whose phosphorylation may be essential for promoting sister chromatid mono-orientation in meiosis I. Identifying these targets is therefore crucial for uncovering the molecular mechanisms governing accurate meiotic chromosome segregation. We further hypothesize that Hrr25 must physically associate with its substrates at the kinetochore to exert its kinase activity. To test this, we employed a yeast two-hybrid (Y2H) screening approach using Hrr25 as bait to identify interacting proteins from a yeast genomic library. Candidate interactors were subsequently analyzed through structural modeling and in silico prediction of phosphorylation sites. This integrative approach enabled the identification of potential Hrr25-interacting proteins that may represent functionally relevant kinase interactors and offer new insights into the regulation of meiotic chromosome segregation.

## 2. Results

### 2.1. Recombinant HRR25 Bait Plasmid Complemented Temperature Sensitivity in hrr25-ts Yeast

**Figure 1:**
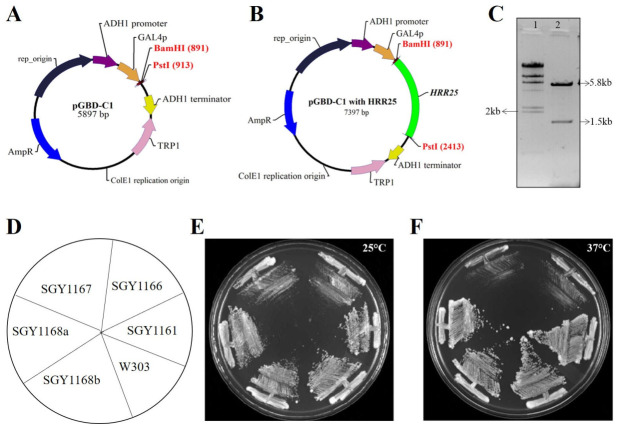
Construction and functional validation of bait vector. Cloning of *HRR25* in pGBD-C1 (**A**–**C**). The vector map of pGBD-C1 (**A**), Vector map of pGBDC1 with cloned *HRR25* ORF (**B**). Cloned fragment is shown in green color. RE digestion of clone with *Bam*HI and *Pst*I (**C**). Lane 1, λ *Hin*dIII marker DNA, Lane 2, digested product. pGBD-C1 carrying *HRR25* ORF complemented the growth in *hrr25-ts* mutant (**D**–**F**). Position of strains on plate (**D**). SGY1168 a and b represent two different clones. Cells were streaked on YPD plates with appropriate controls and incubated at 25 °C (**E**) and 37 °C (**F**). The photograph was taken after two days of growth.

The *HRR25* open reading frame (ORF) was PCR-amplified from wild-type *S. cerevisiae* genomic DNA using primers MA53 and MA54 (Table S3), and cloned into the pGBD-C1 vector using *BamHI* and *PstI* restriction sites. The resulting recombinant plasmid, designated pGBDC1-*HRR25* (pSGB96), was validated by restriction enzyme digestion, which produced the expected ~1.5 kb *HRR25* insert fragment along with the vector backbone (Figure 1A–C).

To confirm functional expression of the bait construct, a complementation assay was performed using a temperature-sensitive *hrr25-ts* mutant strain. This strain (SGY1166) grows optimally at 25 °C but exhibits impaired or no growth at 37 °C. Transformation of the *hrr25-ts* mutant with pGBDC1-*HRR25* (SGY1168a, b) successfully restored growth at the non-permissive temperature of 37 °C, indicating that the cloned *HRR25* gene is functionally expressed. In contrast, cells transformed with the empty vector (SGY1167) or left untransformed (SGY1166) failed to grow under these conditions, confirming the specificity of the complementation (Figure 1D–F).

### 2.2. Yeast Two-Hybrid Library Screening Resulted in Three Putative Interactors of Hrr25

**Figure 2:**
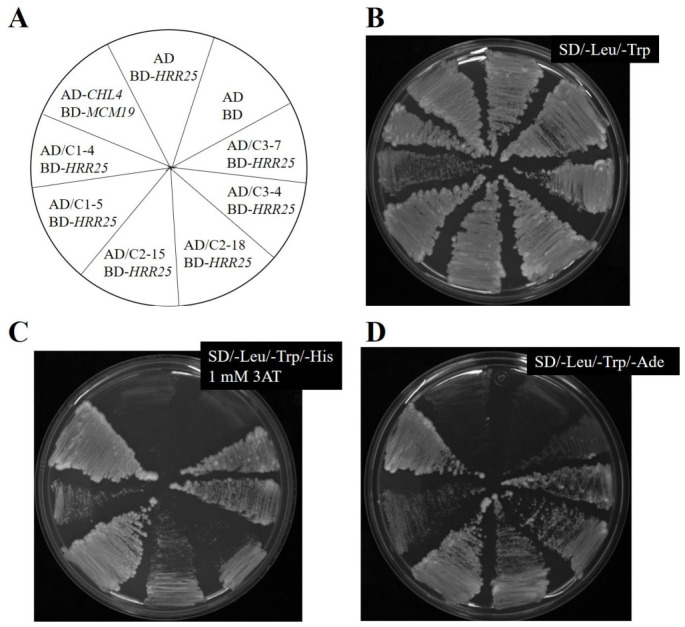
Reporter assay for the putative interactors. A schematic diagram showing the name and position of strains on the plate (**A**). AD-*CHL4* BD-*MCM19* was taken as a positive control. AD BD-*HRR25* and AD BD were used as a negative control. C1, C2 and C3 denote transformants of Y2HL-C1, Y2HL-C2 and Y2HL-C3 libraries. Cells were streaked on an SD/-Leu/-Trp plate (**B**), an SD/-Leu/-Trp/-His dropout plate with 1 mM 3AT (**C**), and an SD/-Leu/-Trp/-Ade plate (**D**).

The host yeast strain PJ69-4A carrying bait plasmid pSGB96 and three dependent Y2H libraries were screened individually. A known protein–protein interaction between kinetochore proteins Chl4 and Mcm19 [17] was used as a positive control. The AD-Chl4 and BD-Mcm19 combination activated all three reporters, whereas negative controls (empty vectors) showed no reporter activity (Figure 2). Across all three libraries, 19 transformants tested positive for all three reporters, SD/-Trp/-Leu/-His with 1 mM 3-AT, SD/-Trp/-Leu/-Ade, and β-Gal assays. These positive clones were subjected to plasmid recovery as described in Section 4. Prey plasmids were isolated, and inserts were analyzed by restriction enzyme digestion. Clones containing inserts larger than 500 bp were selected for further analysis, yielding six candidates. Of these, six clones exhibiting strong reporter activation (Figure 2) were sequenced using a primer specific to the GAL4 activation domain region of the vector. Sequence data were obtained only from three clones and used to perform BLAST (https://blast.ncbi.nlm.nih.gov/Blast.cgi, accessed on 29 April 2026) searches against the *S. cerevisiae* genome database. The resulting genomic coordinates and corresponding ORFs are listed in Table 1. Sequence analysis revealed that C2-15 contained a single ORF (*REP1*), while C3-7 contained two ORFs (*CYR1* and *SYS1*), with Sys1 in the opposite orientation. Clone C1-5 contained three ORFs, *HED1*, *DAD1*, and *YDR015C*, of which only *HED1* was fused in the correct orientation to the activation domain.

### 2.3. Restriction Mapping Revealed a Full-Length DAD1 ORF Within the C1-5

**Figure 3:**
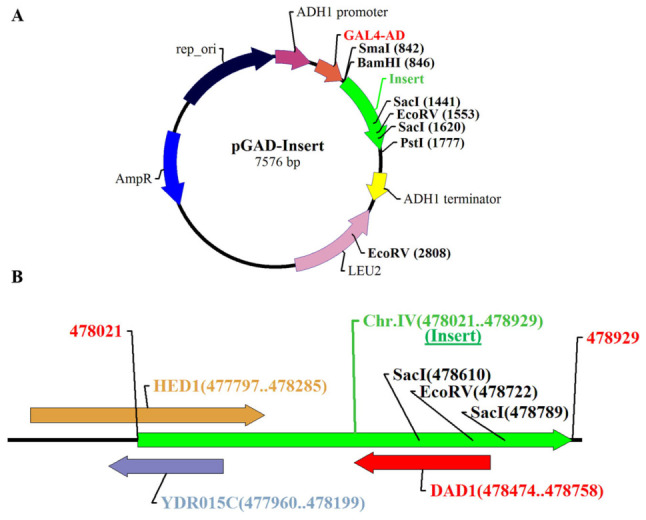
Restriction mapping of the insert present in C1-5. (**A**) Vector map of pGAD-C1 plasmid carrying insert. The insert is shown in green. The position of restriction enzymes is marked on the insert. (**B**) Linearized restriction map of the insert (**B**). Position of ORFs and their coordinates are shown. Each ORF is represented in a different color.

To determine the complete insert composition of clone C1-5, restriction mapping was performed (Figure 3A). This analysis revealed that the insert contained truncated ORFs of *HED1* and *YDR015C*, as well as a full-length *DAD1* ORF (Figure 3B). While *HED1* was confirmed to be fused in-frame with the GAL4 activation domain, both *DAD1* and *YDR015C* were oriented oppositely. Notably, the *DAD1* insert encompassed the entire coding sequence, including upstream regions, and was therefore retained for subsequent structural and functional analyses despite its orientation in the Y2H construct.

### 2.4. Structural Modeling Identified the Phosphorylation Sites on Putative Target Proteins

Further phosphorylation sites were predicted on the putative Hrr25 target proteins. To identify conserved serine (S) and threonine (T) residues likely to be phosphorylated by Hrr25, we employed multiple bioinformatic tools for phosphorylation site prediction. S/T residues consistently predicted by at least two tools were considered potential Hrr25 phosphorylation sites. To prioritize high-confidence candidates, we examined the spatial proximity and orientation of these predicted residues within Hrr25 target protein complex models generated using AlphaFold 3.0.

Based on structural analysis, including distance and positioning, several predicted sites were identified as high-confidence phosphorylation targets (listed in Table 2). For completeness, proteins encoded by ORFs in opposite orientation, including Cyr1, Dad1, and YDR015C, were retained in the analysis to determine whether structural modeling could nonetheless reveal spatially favorable, potentially functional phosphorylation sites.

**Figure 4:**
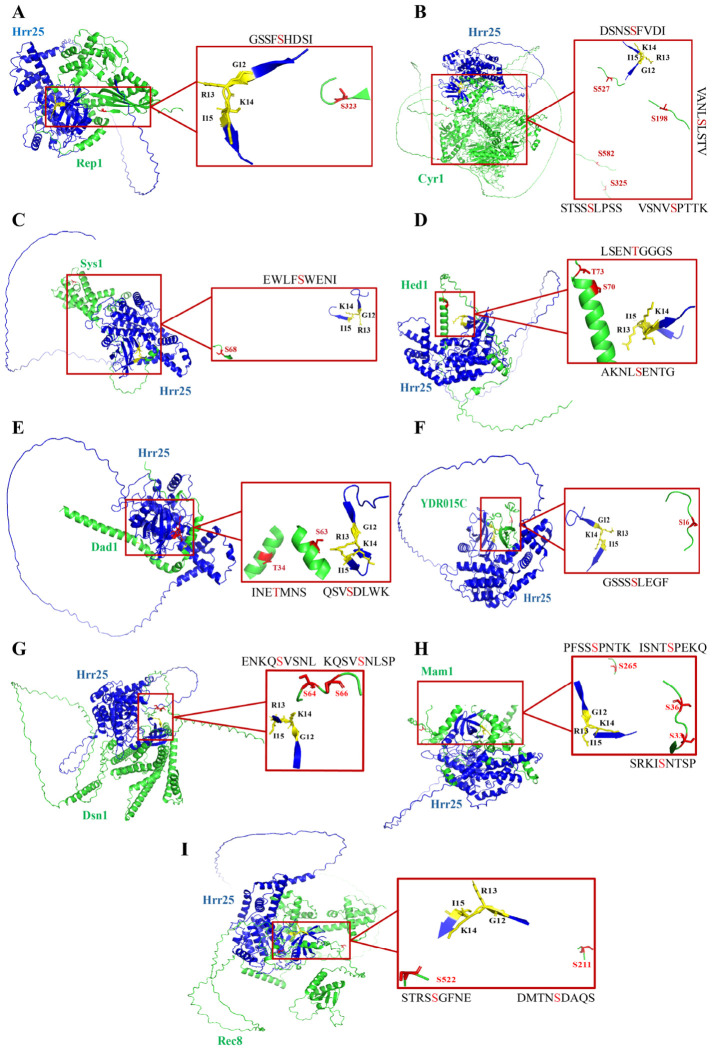
Structural model showing predicted phosphorylation sites on potential Hrr25-interacting target proteins. Panels (**A**–**I**) display the AlphaFold 3.0 predicted complexes of Hrr25 (blue) with its putative target proteins (green): (**A**) Rep1, (**B**) Cyr1, (**C**) Sys1, (**D**) Hed1, (**E**) Dad1, (**F**) YDR015C, (**G**) Dsn1, (**H**) Mam1, and (**I**) Rec8. Predicted phosphorylation sites on the target proteins are shown in red. Insets zoom in on the interaction interface, illustrating the spatial arrangement of key catalytic residues in Hrr25 (yellow) relative to the predicted phosphorylatable serine/threonine residues (red) on the targets.

We identified two high-confidence phosphorylation sites each on Hed1 and Cyr1, and one site each on Dad1 and Rep1. In Hed1, both S70 and T73 were consistently predicted and found in close proximity, suggesting their accessibility for phosphorylation by Hrr25. On Dad1, S63 was appropriately positioned and oriented for potential phosphorylation, whereas T34 appeared distant and poorly oriented, making phosphorylation by Hrr25 less likely. For Cyr1, two high-confidence sites, S198 and S527, were identified. Although additional sites, such as S325 and S582, were predicted to have strong scores by three tools, their spatial positions were unfavorable for Hrr25-mediated phosphorylation. Rep1 contained one high-confidence site, S323. Several of these high-confidence sites, including T73 on Hed1, S198 and S527 on Cyr1, and S323 on Rep1, were located in surface-exposed loops or flexible regions, which could facilitate access by Hrr25. In contrast, the predicted sites on Sys1 and YDR015C were found to be spatially distant or poorly oriented, making them unlikely targets for Hrr25 phosphorylation (Figure 4).

For comparison, we also analyzed previously reported meiotic phosphorylation targets, including Dsn1, Mam1, and Rec8 (Section 1). Structural modeling of these proteins in complex with Hrr25 identified multiple high-confidence phosphorylation sites: S64 and S66 on Dsn1, S33, S36, and S265 on Mam1, and S211 and S522 on Rec8 (Table 2). Consistent with our findings for the candidate targets, these residues were predicted by multiple tools and were located in structurally favorable, surface-accessible regions (Figure 4).

### 2.5. Reporter Expression Was Found Linked to the Presence of Library Plasmid in C1-5

**Figure 5:**
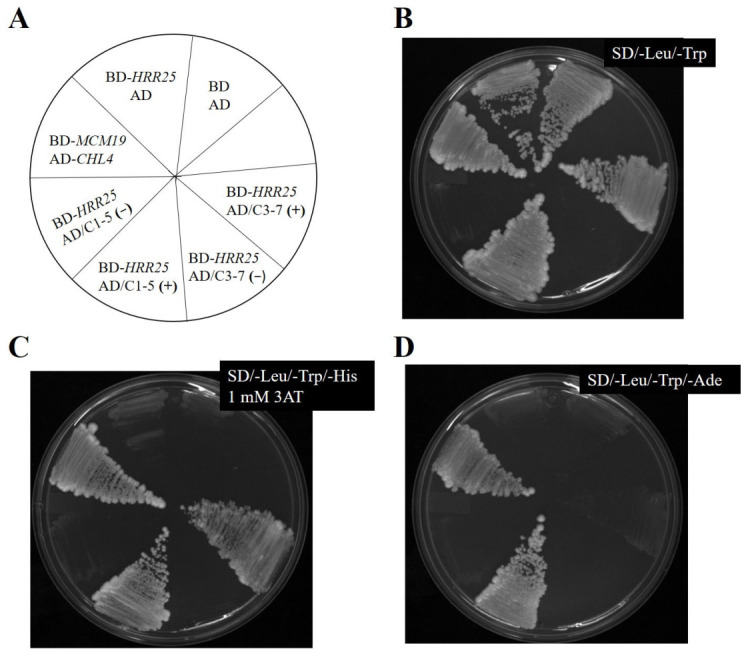
Plasmid-dependent assay from transformants, C1-5 and C3-7. A schematic diagram showing the name and position of strains on the plate (**A**). AD-*CHL4* BD-*MCM19* was taken as a positive control. AD BD-*HRR25* and AD BD were used as a negative control. Transformants C1-5 and C3-7 were tested under both prey plasmid-loss (−) and retransformation (+) conditions. Cells were streaked on Leu and Trp dropout plate (**B**), SD/-Leu/-Trp/-His with 1 mM 3 AT plate (**C**), and SD/-Leu/-Trp/-Ade plate (**D**).

To further validate the positive interaction between Hrr25 and the putative target proteins, a plasmid-dependent assay was performed as described in Section 4. Our sequence analysis of the encoded proteins revealed that clone C2-15 carried the *Rep1* protein, which originates from the 2-micron plasmid (Table 1). Because Rep1 is non-chromosomal and unlikely to be involved in chromosome segregation, C2-15 was excluded from further investigation. Only transformants C1-5 and C3-7 were used in subsequent experiments. The colony obtained from C1-5 transformant, bearing the pGBD-*HRR25* plasmid but lacking the prey plasmid (Trp^+^ Leu^−^), did not show any reporter assay and behaved similarly to the negative control (Figure 5). However, when this strain was re-transformed with the prey plasmid (Trp^+^ Leu^+^), reporter expression including β-Gal assay was restored, indicating a rescued interaction (Figure 5). In contrast, the C3-7 transformant failed to activate the adenine reporter in the plasmid-dependent assay (Figure 5D) suggesting weaker or non-specific interaction. Positive control strains carrying pAD-Chl4/pBD-Mcm19 were streaked on all plates and consistently showed reporter activity, validating the assay conditions.

## 3. Discussion

Given that Hrr25 kinase activity is critical for monopolin function at the centromere, this study aimed to identify potential Hrr25 interactors to elucidate the mechanism underlying chromosome segregation during meiosis I. To achieve this, we employed a Y2H system in conjunction with genetic approaches to screen for Hrr25-interacting proteins.

Y2H genomic libraries in all three reading frames were amplified with high transformation efficiencies. The functional integrity of the BD-Hrr25 fusion protein was confirmed via complementation assays, and the screening system was validated using established positive and negative controls. Following restriction mapping and size selection (>500 bp), six candidates were prioritized, of which three yielded high-quality sequence data. While this size-based filtering enriched for informative inserts, it may have excluded smaller yet functionally relevant interaction domains. The clones that failed to produce reliable sequencing data are likely due to insert instability or structural constraints affecting plasmid quality. Based on Y2H results, structural modeling, biological relevance, and plasmid-dependent validation, clone C1-5 was selected for further analysis.

### 3.1. Is Hrr25-Mediated Phosphorylation of Hed1 and Dad1 Important for Meiosis I?

Among the candidates identified in clone C1-5, Hed1 emerged as a particularly interesting interactor. Hed1 is a meiosis-specific regulator of homologous recombination that inhibits Rad51 [18], thereby promoting crossover formation and ensuring accurate homolog segregation during meiosis I. Previous studies have shown that Hed1 is phosphorylated by the meiosis-specific kinase Mek1 at multiple N-terminal residues [19]. Our structural and in silico analyses further identified S70 and T73 as accessible residues that may serve as potential phosphorylation targets of Hrr25. Notably, both Hrr25 and Hed1 are active during prophase I, suggesting a temporal window for regulatory interaction.

**Figure 6:**
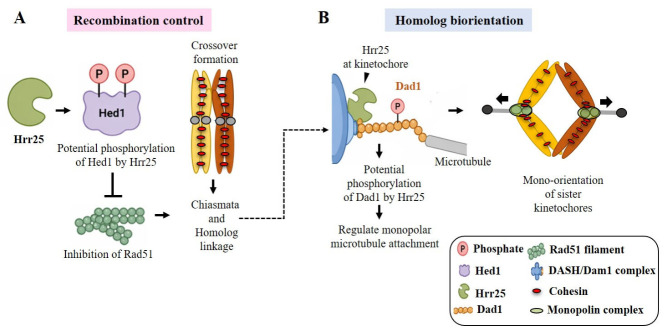
Putative model illustrating a potential interaction of Hrr25 with Hed1 and Dad1 during meiosis I in *S. cerevisiae*. (**A**) Hrr25-mediated phosphorylation of Hed1 fine-tunes homologous recombination during prophase I, ensuring proper crossovers and chiasmata formation. (**B**) At the kinetochore, Hrr25 phosphorylates Dad1, promoting oligomerization/stabilization of the Dam1 complex around the microtubule. Together, these coordinated events support sister kinetochore mono-orientation, bioorientation of homologous chromosomes, and their accurate segregation from metaphase I to anaphase I.

We therefore hypothesize that Hrr25-mediated phosphorylation of Hed1 could provide a functional link between recombination control and kinetochore-driven chromosome segregation. By modulating Hed1 activity, Hrr25 could influence crossover formation and facilitate proper homolog bi-orientation on the meiosis I spindle (Figure 6A). Although this interaction has not been previously reported, the identification of Hed1 in our screen, together with structurally favorable phosphorylation sites, supports a plausible functional connection that warrants further investigation.

We also identified Dad1 as a candidate of interest. Although Dad1 was recovered in the opposite orientation relative to the GAL4 activation domain, precluding its classification as a direct interactor in the Y2H assay, it was retained due to its full-length representation, structural features, and established role at the kinetochore. Dad1 is an essential component of the DASH/Dam1 complex, which mediates kinetochore–microtubule attachment during chromosome segregation [20]. The Dam1 complex is extensively regulated by phosphorylation, primarily through kinases such as Ipl1, Mps1, and Cdk1 [21,22,23,24]. Although direct phosphorylation of Dad1 has not been conclusively demonstrated, the DAD1^E50D^ mutation disrupts meiosis I chromosome segregation and lies within a conserved region predicted to be targeted by Hrr25 [25]. Consistent with this, our analysis identified S63 as a structurally favorable phosphorylation site. Given the proximal localization of Hrr25 and Dad1 at the kinetochore, we propose that Dad1 represents a plausible candidate for Hrr25-mediated regulation (Figure 6B). However, this hypothesis remains speculative and requires experimental validation, including pairwise Y2H assays with full-length constructs, co-immunoprecipitation, in vitro kinase assays, and phospho-mutant analyses.

### 3.2. Limitation and Context-Dependent Detection of Hrr25 Interactions in Y2H Screens

It is important to note that we do not exclude the possibility that additional physical interactors of Hrr25 remained undetected due to limitations of the Y2H system or aspects of our experimental design. Curated interaction databases (e.g., Saccharomyces Genome Database) report over 200 physical interactors of Hrr25, identified through diverse methodologies, whereas only a small subset (~7) have been identified using previous Y2H approaches, including Dcp1, Haa1, Mam1, Pfs1, Pin4, Puf3, and Rad51 [26,27,28,29,30,31,32]. Among these, interactions with Haa1, Pin4, and Puf3 were identified through targeted pairwise Y2H assays, whereas others (e.g., Dcp1, Pfs1, and Rad51) were detected in large-scale, high-throughput screens, where multiple proteins were systematically used as bait. In such genome-wide approaches, it is difficult to determine the specific subset of interactors identified using Hrr25 as bait.

Large-scale Y2H datasets often exhibit limited overlap [29], reflecting differences in experimental design, library composition, and screening conditions. Importantly, to our knowledge, this study represents the first systematic Y2H screen using Hrr25 as bait against a yeast genomic library.

Furthermore, many interactions are likely context-dependent. For example, during meiosis I, Mam1 recruits Hrr25 to kinetochores, positioning it in proximity to substrates such as Dsn1 [14]. Such interactions may not be recapitulated in the Y2H system, which lacks appropriate cofactors, post-translational modifications, and cellular context. Additionally, known substrates such as Dsn1 and Rec8 have not been identified in previous Y2H screens, despite strong evidence for their phosphorylation. Consistent with this, our structural modeling also supports accessible phosphorylation sites on these proteins, underscoring that kinase–substrate relationships may not always be captured by interaction-based assays. Therefore, the absence of previously reported interactors in our dataset does not necessarily indicate false negatives; rather, it highlights the system’s limitations and the need for complementary approaches.

We also note that our phosphorylation predictions based on AlphaFold 3.0 structural models should be interpreted with caution. While AlphaFold provides highly accurate static structural predictions, it does not capture the dynamic conformational changes that occur during enzyme–substrate interactions. These dynamics may influence residue accessibility and orientation, potentially affecting phosphorylation outcomes. Future studies incorporating molecular dynamics simulations and experimental validation will be necessary to refine these predictions and better understand phosphorylation site accessibility in vivo.

## 4. Materials and Methods

### 4.1. Yeast Strains, Bacterial Strains, Plasmids, and Primers

The parent yeast strain W303 (*MATa*, *ura3-52*, *lys2-801*, *ade2-101*, *trp1-Δ63*, *his3-Δ200*, *leu2-Δ1*) was used to construct all experimental strains, as specified in Table S1. All the bacterial strains used in this study are listed in Table S2. The Y2H plasmids used were pGBDC1 and pGADC1 [33], and the positive control plasmids used were pAD-Chl4 and pBD-Mcm19 [17]. The primers used in this study are listed in Table S3.

### 4.2. Growth Media and Growth Conditions

Yeast strains were cultured on YPD medium (1% yeast extract, 2% peptone, 2% dextrose) at 30 °C unless otherwise specified. For screening transformants, synthetic dropout (SD) media containing yeast nitrogen base (YNB), dextrose, and a customized synthetic complete amino acid mix lacking one or more specific nutrients, as required by the experiment, were used. Bacterial strains, either transformed with plasmids or untransformed, were grown in Luria–Bertani (LB) broth or on LB agar plates at 37 °C.

### 4.3. Construction of Recombinant Plasmids

Plasmid DNA isolation, preparation of competent *E. coli* cells, and bacterial transformation procedures were carried out manually [34]. To construct the recombinant bait plasmid, total genomic DNA was isolated from yeast cells and used as a template to PCR amplify the *HRR25* gene. Specific restriction enzyme restriction sites, *BamH*1 and *Pst*1, were incorporated into the forward and reverse primers. The plasmid vector DNA, pGBDC1, was isolated from *E. coli*, digested with the same restriction enzymes used for the PCR product, and subsequently ligated to the *HRR25* insert. The ligation mixture was transformed into competent *E. coli* cells. Transformants were screened using a combination of assays including agarose gel electrophoresis to assess plasmid size shift (retardation assay), restriction enzyme digestion, and PCR amplification of the insert.

### 4.4. Amplification and Quality Assessment of Yeast Two-Hybrid Library DNA

Y2H libraries carried in the *pGAD* vector [33], representing three different reading frames (Y2HL-C1, Y2HL-C2, Y2HL-C3), were amplified. Competent *E. coli* DH5α cells were individually transformed with each *pGAD* vector carrying a library in a specific reading frame. The transformed cells were cultured, and plasmid DNA was extracted using a maxi-prep protocol to amplify the libraries.

To assess the quality and efficiency of the amplified libraries, a small aliquot of each plasmid preparation was transformed into competent *E. coli* cells. Plasmid DNA was then isolated from at least 10 individual colonies per library and subjected to restriction enzyme digestion using *SmaI* and *PstI*. Library efficiency was calculated as the percentage of plasmids containing an insert relative to the total number analyzed. The observed efficiency was approximately 80% for Y2HL-C1 and Y2HL-C2, and 85% for Y2HL-C3. To ensure comprehensive coverage, the amplified Y2H library should represent the yeast genome approximately three times. The number of yeast colonies required for screening was calculated using the following formula:

Number of colonies to be screened = 3 × Yeast genome size/Average insert size

Using this formula and accounting for transformation efficiency, the total number of colonies screened was 45,375 for Y2HL-C1 and C-2 and 42,471 for Y2HL-C3. The average insert size for these libraries was ~1 kb.

### 4.5. Yeast Two-Hybrid Studies

Y2H analyses were performed using the *S. cerevisiae* strain PJ69-4A, which carries three reporter genes, *HIS3*, *ADE2*, and *LacZ*, used to detect protein–protein interactions [33]. Plasmids pGBD and pGAD, with or without gene fusions, were transformed into PJ69-4A. For library screening, the PJ69-4A strain harboring the bait construct (pGBD vector with *TRP1* marker and HRR25 gene insert) was transformed with a *S. cerevisiae* genomic two-hybrid library cloned into a pGAD vector carrying the *LEU2* selection marker. Transformants were selected on SD plates lacking leucine and tryptophan (SD/-Leu-Trp) to maintain both plasmids.

Positive interactors were first screened by patching colonies onto SD/-Leu-Trp-His plates supplemented with 1 mM 3-amino-1,2,4-triazole (3-AT) to suppress background *HIS3* expression. Colonies that grew on this medium were further tested on SD/-Leu-Trp-Ade plates. Those showing growth on both selective media were subjected to β-galactosidase (β-Gal) assays to confirm *LacZ* reporter activation.

### 4.6. Colony-Lift Filter Assay for β-Galactosidase Activity

β-Galactosidase activity was evaluated qualitatively by color development on filters. Yeast colonies were patched onto SD selection plates alongside appropriate positive and negative control strains. After full colony growth, a circular nitrocellulose (NC) filter matching the Petri dish size was gently placed over the colonies and allowed to sit for 2–3 min to transfer cells. The NC filter with adhered colonies was carefully removed and placed in a separate Petri dish, then rapidly frozen using liquid nitrogen poured directly onto the filter. Once frozen, the filter was transferred to a fresh Petri dish pre-lined with Whatman filter paper. An aliquot of Z buffer (containing Na_2_HPO_4_·7H_2_O, NaH_2_PO_4_, KCl, MgSO_4_·7H_2_O, β-mercaptoethanol, and distilled water) supplemented with X-gal at a final concentration of 1 mg/mL was gently added from the edge to saturate the NC filter. The setup was incubated at 30 °C for 1–2 h. Colonies exhibiting a visible blue or purple color change were considered positive for β-galactosidase activity.

### 4.7. Recovery of Plasmid DNA from Yeast Cells

To recover library plasmids from yeast, total genomic DNA was isolated from a 5 mL overnight-grown yeast culture. The DNA was resuspended in 50 µL of TE buffer. An aliquot of 10 µL was then transformed into competent *E. coli* KC8 cells. Transformants were selected on LB agar plates supplemented with ampicillin. Plasmid DNA was subsequently isolated from bacterial colonies and analyzed for the presence of inserts via restriction enzyme digestion.

### 4.8. Plasmid-Dependent Yeast Two-Hybrid Assay

To confirm that reporter activation was dependent on both bait and prey plasmids, a plasmid-dependent assay was performed. A library-derived yeast transformant that tested positive for all three reporter genes (*HIS3*, *ADE2*, and *LacZ*) was selected. This strain, containing both the bait (*TRP1* marker) and prey (*LEU2* marker) plasmids, was initially grown on SD/-Leu/-Trp medium and then cultured in YPD broth for approximately 40 generations to allow plasmid segregation. Following growth, cells were streaked onto YPD plates to isolate single colonies. Approximately 40–50 single colonies were patched onto YPD, SD/-Leu, and SD/-Trp plates. All colonies were expected to grow on YPD. Colonies that showed growth on SD/-Trp but failed to grow on SD/-Leu were identified as having retained only the bait plasmid (Trp^+^, Leu^−^) and lost the prey plasmid. These selected colonies, now lacking the prey plasmid, were expected to lose reporter activity, thereby confirming that interaction-driven reporter activation required both plasmids. Reintroduction of the prey plasmid into these strains should restore reporter activity, further validating the interaction.

### 4.9. Bioinformatic Analyses of Potential Hrr25 Phosphorylation Sites on Target Proteins

*S. cerevisiae* specific bioinformatic tools (NetPhosYeast 1.0 [35], GPS 6.0 [36]) and non-species-specific (NetPhos-3.1 [37], PhosphoSVM [38]) were used for sequence-based phosphorylation site predictions. For both species specific and non-specific tools used in this study, the target sites were selected and sorted for Casein Kinase 1 (CK1) and the parameters were kept as default. The amino acid sequences for the Hrr25 and potential target proteins were retrieved from the *Saccharomyces* genome database (SGD): Hrr25 (SGD: S000006125), Hed1 (SGD: S000113613), Cyr1 (SGD: S000003542), YDR015C (SGD: S000002422), Rep1 (SGD: S000029675), Sys1 (SGD: S000003541), Dad1 (SGD: S000002423), Dsn1 (SGD: S000001449), Rec8 (SGD: S000006211), and Mam1 (SGD: 000000908). The FASTA sequences of the target proteins were used as the inputs for phosphorylation site analyses. The outputs from all the tools were manually compared to identify the common phosphorylation sites on Hrr25 target proteins.

Due to the unavailability of complex structures in the Protein Data Bank (PDB), full-length amino acid sequences of Hrr25 and each target protein were retrieved from SGD and submitted as a two-protein complex prediction job to AlphaFold 3.0 [39] using default parameters. The top-ranked model for each complex, selected based on ipTM score, was used for structural analyses in PyMOL v3.1.4.1.

Residues predicted by at least two independent tools with scores above the default significance threshold for each tool were considered candidate phosphorylation sites. High-confidence sites were further defined as those whose predicted residues were spatially accessible (based on proximity and orientation) and located in surface-exposed or flexible regions.

## 5. Conclusions

Overall, this study provides preliminary but compelling evidence for potential Hrr25 interactors. The identification of Hed1 suggests a possible link between kinase activity and recombination regulation, while Dad1 is an intriguing candidate given its established role in kinetochore function and chromosome segregation during meiosis I. Further biochemical and genetic investigations will be essential to validate these interactions and elucidate their functional significance. Collectively, these findings lay the groundwork for future studies to uncover novel mechanisms governing chromosome segregation during meiosis.

## Figures and Tables

**Table 1:** Summary of BLAST result from query sequences against *Saccharomyces cerevisiae* genome.

Name of Clone	Identified Coordinates from the BLAST Result	Identified ORFs Within the Coordinates	Homology (bps)
C1-5	478,021…478,579	*HED1*(Chr. IV 477,797…478,285)	266
		*DAD1*(Chr IV 478,474…478,758)	108
		*YDR015C* (Chr. IV 477,960…478,199)	180
C2-15	1606…2531	*REP1* (1887…3008)	648
C3-7	430,767…431,361	*CYR1* (Chr X 425,157…431,237)	474
		*SYS*1 (Chr X 431,589…432,200)	15

**Table 2:** The table summarizes the predicted phosphorylation sites and high-confidence target residues for each listed protein.

Protein	Predicted Phosphorylation Residues	High-Confidence Target Residues
Hed1	S70, T73	S70, T73
Dad1	T34, S63	S63
YDR015C	S16	Not found
Sys1	S68	Not found
Cyr1	S198, S325, S527, S582	S198, S527
Rep1	S323	S323
Dsn1	S64, S66, S418, S547	S64, S66
Mam1	S8, S29, S33, S36, S265, T35	S33, S36, S265
Rec8	S179, S211, S314, S436, S522, S633	S211, S522

## Data Availability

The original contributions presented in this study are included in the article/Supplementary Material. Further inquiries can be directed to the corresponding author.

## Supplementary Materials

The following supporting information can be downloaded at: https://www.mdpi.com/article/10.3390/ijms27094083/s1.
