# Supplementary material for: Using Yeast Two-Hybrid Screening and Structural Modeling to Identify Candidate Hrr25 Kinase Interactors at the Meiotic Kinetochore in Saccharomyces cerevisiae
Source: Int J Mol Sci. 2026 May 2;27(9):4083. doi: 10.3390/ijms27094083 (PMC13163615; doi:10.3390/ijms27094083)
Supplement: Supplementary file 1 [file ijms-27-04083-s001.zip › ijms-4190928-supplementary.pdf]

Supplementary information:

Table S1. List of yeast strains constructed in this study.

Strain	Mating type	Genotype
SGY1070	*MAT***a**	*trp1-901 leu2-3, 112 ura3-52 his3-200 gal4Δ gal80Δ Met2::GAL7-lacZ LYS2::GAL1-HIS3 GAL2-ADE2 pGBDC1:: TRP1 + pGAD424::LEU2*
SGY1158	MAT**a**	*trp1-901 leu2-3, 112 ura3-52 his3-200 gal4Δ gal80Δ Met2::GAL7-lacZ LYS2::GAL1-HIS3 GAL2-ADE2 pGBDC1-HRR25::TRP1*
SGY1161	MAT**a**	*trp1-901 leu2-3, 112 ura3-52 his3-200 gal4Δ gal80Δ Met2::GAL7-lacZ LYS2::GAL1-HIS3 GAL2-ADE2 pGBDC1:: TRP1*
SGY1166	*MAT***a**	*ura3Δ0 leu2Δ0 his3Δ1 lys2Δ0 met15Δ0 can1Δ0::LEU2-MFA1pr::HIS3, hrr25-ts::URA3*
SGY1167	*MAT***a**	*ura3Δ0 leu2Δ0 his3Δ1 lys2Δ0 met15Δ0 can1Δ0::LEU2-MFA1pr::HIS3, hrr25-ts::URA3 + pGBDC1:: TRP1*
SGY1168a	*MAT***a**	*ura3Δ0 leu2Δ0 his3Δ1 lys2Δ0 met15Δ0 can1Δ0::LEU2-MFA1pr::HIS3 hrr25-ts::URA3, pGBDC1-HRR25::TRP1*
SGY1168b	*MAT***a**	*ura3Δ0 leu2Δ0 his3Δ1 lys2Δ0 met15Δ0 can1Δ0::LEU2-MFA1pr::HIS3 hrr25-ts::URA3, pGBDC1-HRR25::TRP1*
SGY1428	*MAT***a**	*trp1-901 leu2-3, 112 ura3-52 his3-200 gal4Δ gal80Δ Met2::GAL7-lacZ LYS2::GAL1-HIS3 GAL2-ADE2 pGBDC1-HRR25:: TRP1 + pGAD-C1::LEU2*

Table S2. List of bacterial strains used in this study.

Strain	Genotype
KC8	*E.coli KC8 (US 15) Nx 1486 M+ K-12 leuB-600 trpC 9830 PyrF::Tn-5 hisB 463 del lacx74 Str A galU gal K*
DH5alpha	*fhuA2 Δ(argF-lacZ)U169 phoA glnV44 Φ80 Δ(lacZ)M15 gyrA96 recA1 relA1 endA1 thi-1 hsdR17*

Table S3. List of primers used in this study.

Primer name	Description	Sequence
MA53	Cloning of *HRR25* in pGBD vector (with *BamH*I site)	5’ gagacgcggatcatggacttaagagtaggaag 3’
MA54	Cloning of *HRR25* in pGBD vector (*Pst*I site)	5’ gagaaaaactgcagttacaaccaaattgactggc 3’
